# The dependence of Ni-Fe bioxide composites nanoparticles on the FeCl_2_ solution used

**DOI:** 10.1186/1752-153X-6-127

**Published:** 2012-10-30

**Authors:** Yueqiang Lin, Jian Li, Lihua Lin, Xiaodong Liu, Longlong Chen, Jun Fu

**Affiliations:** 1 School of Physical Science &Technology, MOE Key Laboratory on Luminescence and Real-Time Analysis, Southwest University, Chongqing, 400715, People’s Republic of China

**Keywords:** Composite, Nanoparticles, FeCl_2_ solution, Concentration

## Abstract

**Background:**

Ni_2_O_3_- γ-Fe_2_O_3_ composite nanoparticles coated with a layer of 2FeCl_3_·5H_2_O can be prepared by co-precipitation and processing in FeCl_2_ solution. Using vibrating sample magnetometer (VSM), X-ray diffraction (XRD), transmission electron microscopy (TEM) and X-ray photoelectron spectroscopy (XPS) diffraction techniques, the dependence of the preparation on the concentration of the FeCl_2_ treatment solution is revealed.

**Results:**

The magnetization of the as-prepared products varied non-monotonically as the FeCl_2_ concentration increased from 0.020 M to 1.000 M. The Experimental results show that for the composite nanoparticles, the size of the γ-Fe_2_O_3_ phase is constant at about 8 nm, the Ni_2_O_3_ phase decreased and the 2FeCl_3_·5H_2_O phase increased with increasing concentration of FeCl_2_ solution. The magnetization of the as-prepared products mainly results from the γ-Fe_2_O_3_ core, and the competition between the reduction of the Ni_2_O_3_ phase with the increase of the 2FeCl_3_·5H_2_O phase resulted in the apparent magnetization varying non-monotonically.

**Conclusions:**

When the concentration of FeCl_2_ treatment solution did not exceed 0.100 M, the products are spherical nanoparticles of size about 11 nm; their magnetization increased monotonically with increasing the concentration of FeCl_2_ solution due to the decreasing proportion of Ni_2_O_3_ phase.

## Introduction

Magnetic nanoparticles with diameters less than 100 nm have attracted increasing interest as particles in this size range may allow investigation of fundamental aspects of magnetic ordering phenomena in magnetic materials with reduced dimensions and could lead to new technological applications [1-5]. Studies of magnetic nanoparticles have focused on the development of novel synthetic methods [5]. A nanocomposite is a material composed of two or more phases, one of which has a grain size of less than 100 nm. The combination of different physical or chemical properties may give rise to completely new materials [6,7]. It has been demonstrated that the formation of a passive coating of an inert material on the surface of iron oxide nanoparticles can help to improve their chemical stability and prevent their aggregation in liquids [8-11]. Recently, composite nanoparticles based on magnetic iron oxide have been prepared [12-16]. Such magnetic nanocomposites have applications ranging from ferrofluids to separation science and technology [17].

In previous work, we described a method to prepare magnetic nanoparticles using a chemically induced transition[15,16,18,19] and Ni-Fe bioxide composite nanoparticles were prepared using this method. In the preparation, a precursor consisting of FeOOH wrapped in Ni(OH)_2_ was synthesized by the well-known co-precipitation method. Then, using heat treatment in 0.25 M FeCl_2_ solution at 100°C, a transition took place in which in addition to the Ni(OH)_2_ partially dissolving, the FeOOH/Ni(OH)_2_ precursor was transformed into γ-Fe_2_O_3_/ Ni_2_O_3_ composite nanoparticles coated with FeCl_3_[15]. The Ni_2_O_3_ is weakly ferromagnetic [16] and the FeCl_3_ is paramagnetic. Experiments have shown that such Ni-Fe bioxide composite nanoparticles are very suitable for the synthesis of ferrofluids [20]. This chemically induced transition using FeCl_2_ solution may provide a new route for the preparation of oxide nanoparticles. In the present work, we have investigated the characteristics of Ni-Fe bioxide composite nanoparticles as a function of the concentration of FeCl_2_ treatment solution.

## Experimental

### Preparation

The preparation of the Ni-Fe bioxide composite nanoparticles was divided into two steps. Firstly, the precursor based on FeOOH wrapped with Ni(OH)_2_ was synthesized using the co-precipitation method, which has been described in detail elsewhere [15,21]. The second step was to add the precursor to FeCl_2_ solution, using concentrations of 0.025 M, 0.050 M, 0.075 M, 0.100 M, 0.125 M, 0.250 M, 0.500 M, 0.750 M and 1.000 M, to obtain 400ml of the mixed solution. Then this solution was heated to boiling point for 30 min in atmosphere; the nanoparticles precipitated gradually after the heating had stopped. Finally, these particles were dehydrated with acetone and allowed to dry naturally.

### Characterization

A series of Ni-Fe oxide composite nanoparticles was prepared by a chemically induced transition involving FeCl_2_ solution. The dependence on the concentration of the FeCl_2_ solution was investigated by measuring the specific magnetization curves of the samples at room temperature using a vibrating sample magnetometer (VSM, HH-15, applied field up to 10^4^ Oe).

The samples were prepared using FeCl_2_ solutions 0.025 M, 0.075 M, 0.100 M, 0.125 M and 0.500 M, which were named samples (1), (2), (3), (4) and (5), respectively. In addition to the magnetic measurements, their crystal structures, morphology and chemical composition were analyzed by X-ray diffraction (XRD, XD-2, Cu Kα radiation), transmission electron microscopy (TEM, JEM-2100F, at 100 kV) and X-ray photoelectron spectroscopy (XPS, Thermo ESCA250, Mg target).

## Results and analysis

**Figure 1:**
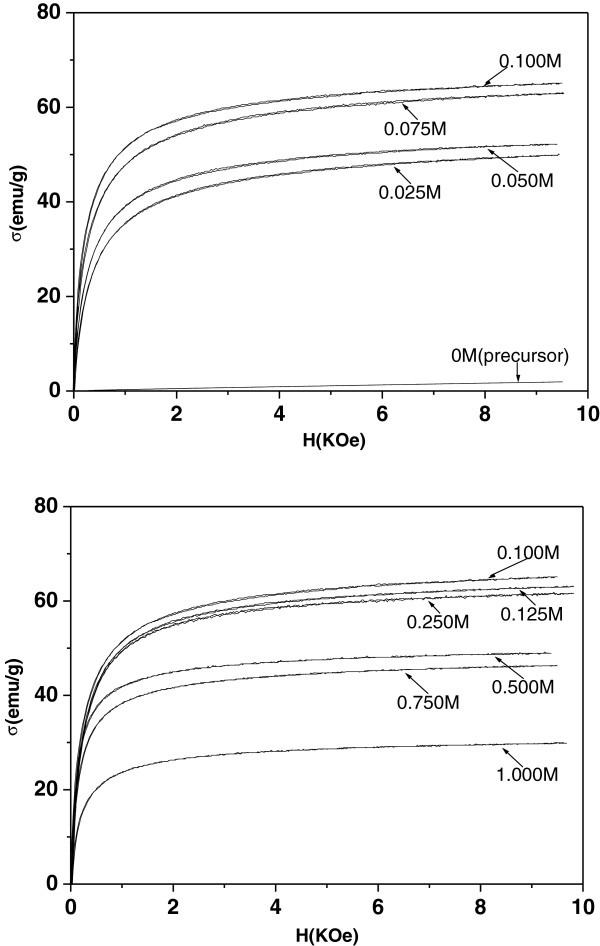
Specific magnetization curves for the samples.

Figure 1 shows the specific magnetization curves of the samples. Clearly, all samples exhibited ferromagnetic features, with their specific magnetization varying non-monotonically with the concentration of FeCl_2_ solution. At first, the magnetization strengthened as the FeCl_2_ concentration increased from 0.025 M to 0.100 M, then the magnetization weakened as the concentration increased from 0.100 M to 1.000 M.

**Figure 2:**
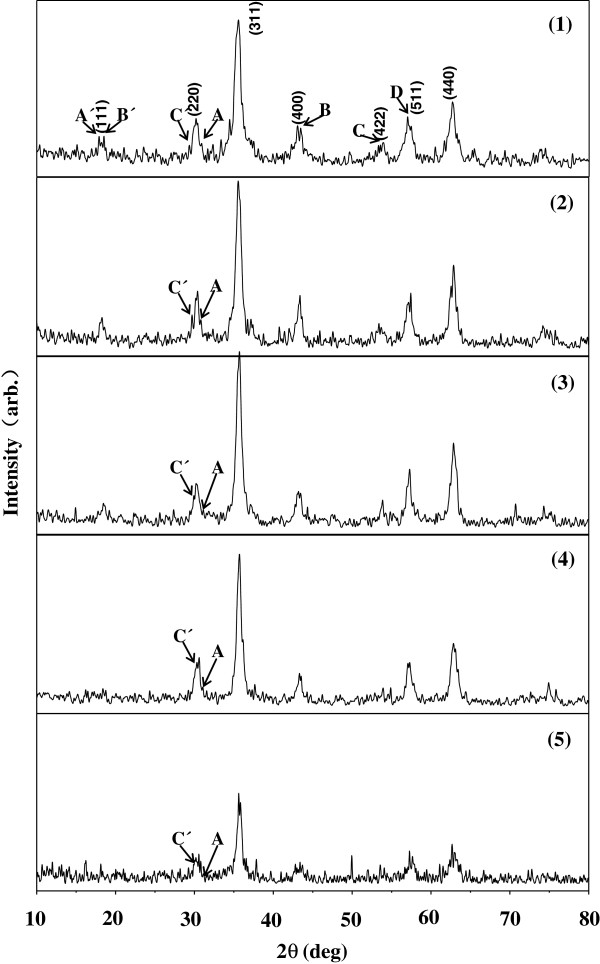
XRD patterns for the samples.

The XRD patterns of the samples are shown in Figure 2. The results show that these samples contain mainly γ-Fe_2_O_3_ with a trace of Ni_2_O_3_ and 2FeCl_3_·5H_2_O, as indicated by the arrows A, B, C and D for Ni_2_O_3_, and by arrows A′, B′ and C′ for 2FeCl_3_·5H_2_O. For the ferrite nanoparticles, the grain sizes d_c_ can be estimated from the half-maximum width of the (311) diffraction peak β using Scherr’s formula [22], d_c_=Kλ/βcosθ, where K is a constant 0.89, λ is the X-ray wavelength (Cu K_α_=0.1542 nm) and θ is the Bragg diffraction angle of the (311) plane. The calculated results gave about the same value 8 nm for the γ-Fe_2_O_3_ grains in all the samples. In addition, comparing the intensity ratios of the A peak of Ni_2_O_3_(d=0.2800 nm) with the C’peak of 2FeCl_3_·5H_2_O (d=0.2980 nm) show that the proportion of Ni_2_O_3_ was reduced and 2FeCl_3_·5H_2_O increased as the concentration of the FeCl_2_ solution increased.

**Figure 3:**
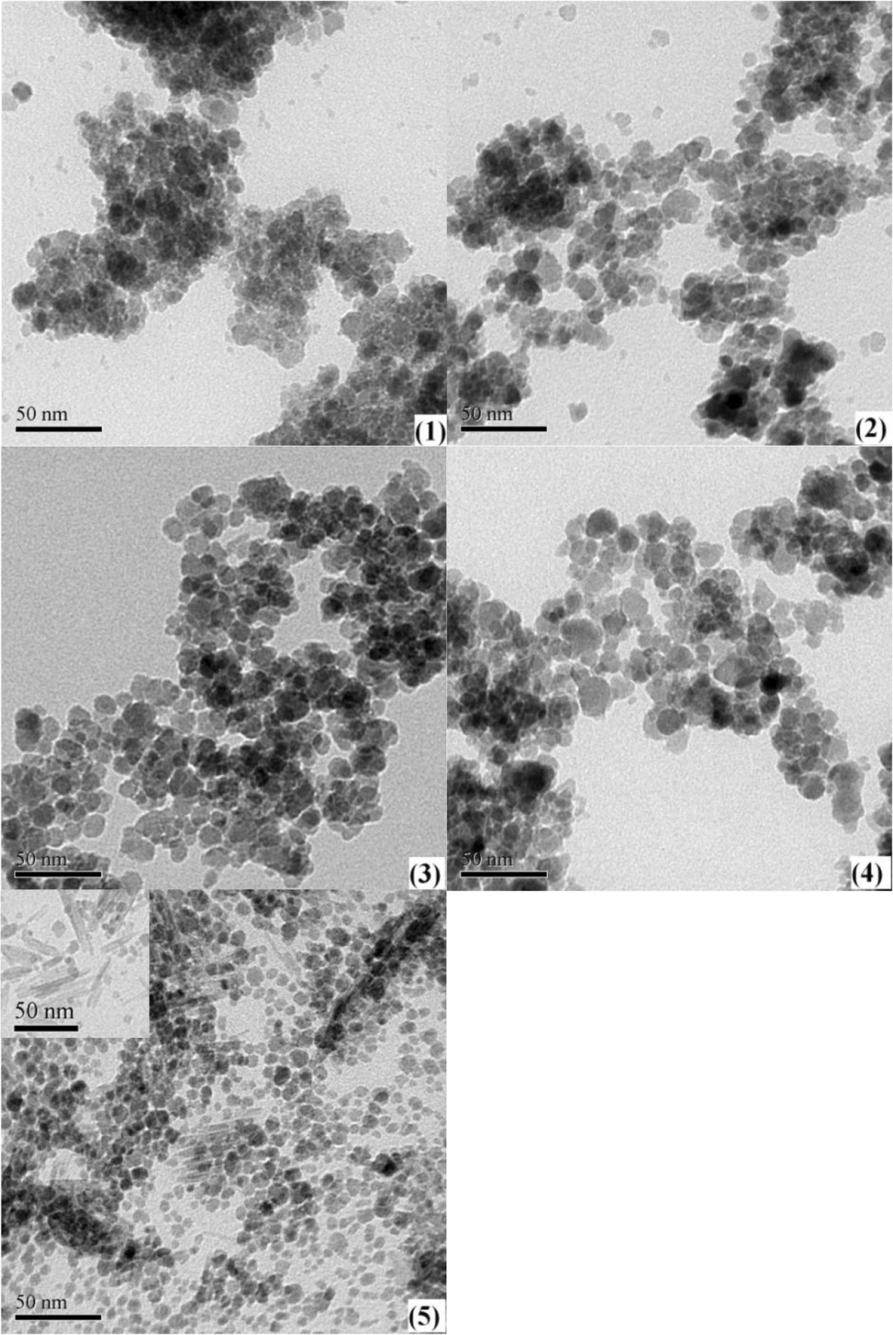
TEM images for the samples.

**Figure 4:**
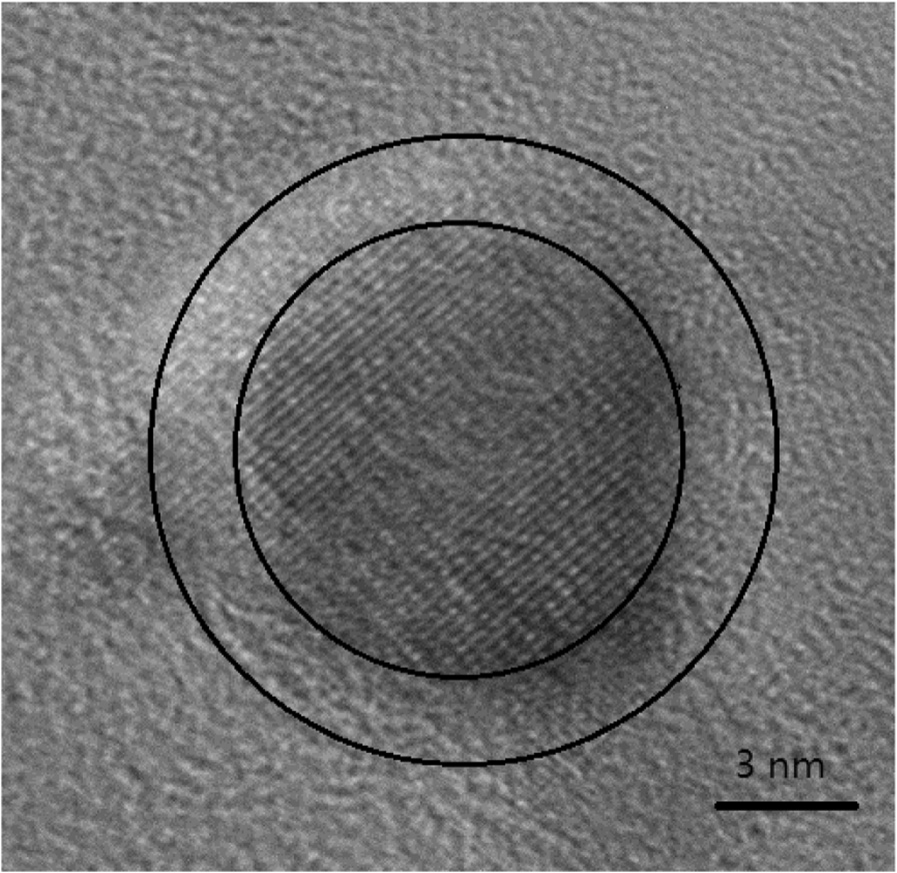
High-resolution TEM image of the particle from sample (3).

TEM observations of the samples are shown in Figure 3. These results show that the particles in samples (1), (2), (3) and (4) are nearly spherical, with an average particles size d_p_ of about 11 nm, but in sample(5) there are a few rod-shaped particles (shown in the insert) in addition to the spherical particles. Clearly, the size of the spherical particles in sample(5) is less than those of samples (1), (2), (3) and (4), and is about 8 nm. High-resolution TEM results reveal that the particles have core-shell structure, as Figure 4 shown.

**Figure 5:**
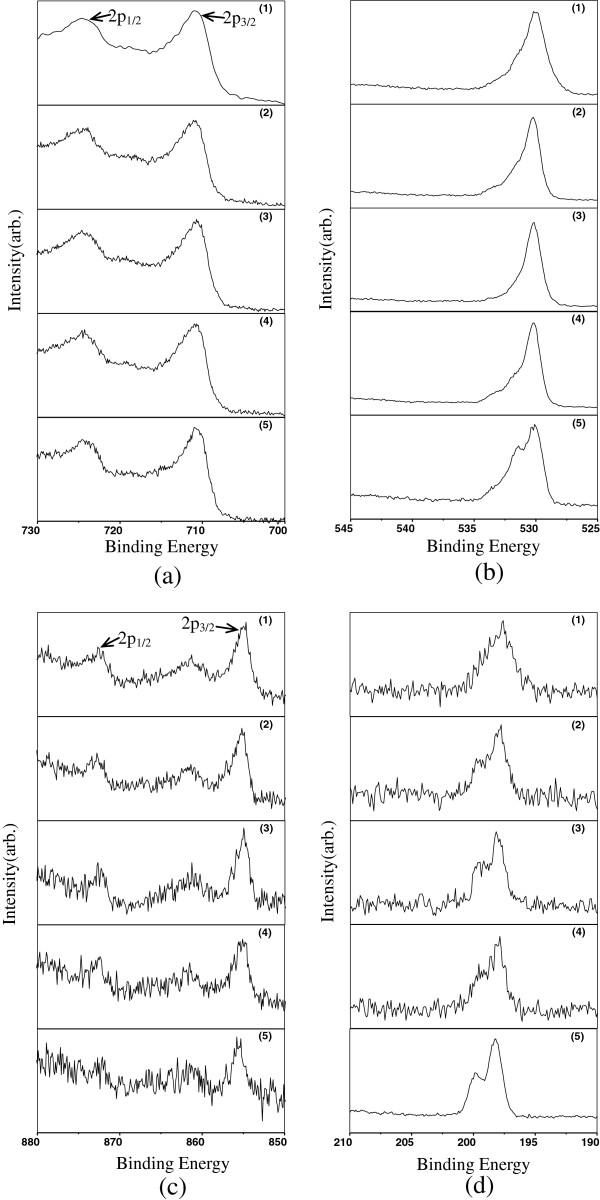
XPS results: Fe2p(a), O1s(b), Ni2p(c) and Cl1s(d).

XPS measurements confirmed that there were Fe, O, Ni and Cl in the samples as illustrated in Figure 5. By analysis of the binding energies in the spectra, it can be deduced that the samples consisted of Fe_2_O_3_, Ni_2_O_3_ and FeCl_3_. The binding energy data are listed in Table 1. A quantitative analysis shows that for samples (1), (2), (3) and (4), the ratio Ni:Cl decreased in that order, the ratio Fe:Ni clearly increased and the ratio Fe:Cl increased slightly. For sample (5), the ratio Fe:Cl was clearly lower than that for sample (4), along with the ratio Ni:Cl, The ratio Fe:Ni was, however, higher. The complete data are listed in Table 2. In conclusion, it can be determined that for all the samples, the proportion of Ni_2_O_3_ phase decreased and FeCl_3_ phase increased as the concentration of FeCl_2_ solution increased. This also agrees with the XRD results.

**Table 1:** Binding energy data for the elements of the samples from XPS(eV)

	**Fe2p**_**3/2**_	**O1s**	**Ni2p**_**3/2**_	**Cl2p**
(1)	710.50	530.04	854.93	197.66
(2)	710.66	530.13	855.10	197.73
(3)	710.36	530.11	854.96	198.08
(4)	710.43	530.04	855.04	197.95
(5)	710.86	530.34	855.66	198.35
Fe_2_O_3_	710.70	529.80		
Ni_2_O_3_		531.80	855.60	
FeCl_3_	711.08			198.72

Note. The standard data for Fe_2_O_3_, Ni_2_O_3_, and FeCl_3_ are taken from the HANDBOOK OF X-RAY PHOTOELECTRON SPECTROSCOPY By C. D. Wanger, W. M. Riggs, L. E. Davis, J. F. Moulder, G. E. Muilenberg (Editor).

**Table 2:** The atomic percentages of Fe, O, Ni and Cl from XPS measurement and the molar ratio of Ni2O3/FeCl3

	**Fe**	**O**	**Ni**	**Cl**	**Fe : Ni : Cl**			Ni_2_O_3_/ FeCl_3_
(1)	13.79	72.50	8.56	5.15	1	0.62	0.37	1/0.40
(2)	15.48	74.94	5.88	4.58	1	0.38	0.30	1/0.53
(3)	19.21	71.06	4.15	5.58	1	0.22	0.29	1/0.89
(4)	16.74	75.45	3.11	4.70	1	0.19	0.28	1/1.02
(5)	18.71	67.21	1.84	12.25	1	0.10	0.65	

## Discussion

The results show that in the preparation of Ni-Fe bioxide nanoparticles, when the concentration of FeCl_2_ solutions were less than 0.5 M, the samples (1), (2), (3) and (4) were single spherical particles consisting of a γ-Fe_2_O_3_ core, Ni_2_O_3_ outside the core and with an outermost layer of 2FeCl_3_·5H_2_O. However, when the concentration reached 0.5 M, sample (5) formed rod-shaped particles together with spherical particles than smaller those of samples (1), (2), (3) and (4).

**Figure 6:**
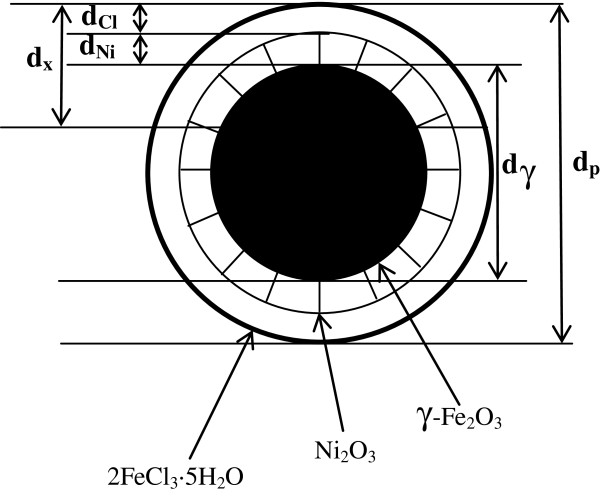
**The schematic cross-section of the particle detected by XPS for the samples (1), (2), (3) and (4).** Note: $d_{x}<\frac{1}{2}d_{p}$.

For samples (1), (2), (3) and (4), the results of both XRD analysis and TEM observations indicated that the γ-Fe_2_O_3_ grain size and the size of the complete particles remain about constant. However, the XPS results showed that the proportion of Ni_2_O_3_ decreased and FeCl_3_ increased smoothly. This suggests that over the XPS detection range d_x_ determined by the mean free paths of the electrons detected [23,24] and which is about 3 nm, the volume fraction of the γ-Fe_2_O_3_ phase remained constant(i.e. diameter of γ-Fe_2_O_3_ core d_r_ can be regarded as constant), as did the sum of the volume fractions of Ni_2_O_3_ and 2FeCl_3_·5H_2_O, whose detection ranges are d_Ni_ and d_Cl_, respectively. So, from samples (1) to (4), the reduction of Ni species and the increase of Cl species in the XPS results corresponds to the variation of the volume fraction, a thinning of the Ni_2_O_3_ layer and a thickening of the 2FeCl_3_·5H_2_O layer. This is also in agreement with the clear increase of the Fe:Ni ratio and the slight increase of the Fe:Cl ratio as the concentration of FeCl_2_ solution increases. Since samples (1), (2), (3) and (4) consist of spherical particles and the measured XPS depth d_x_ is greater than the combined thickness of Ni_2_O_3_ (d_Ni_) and FeCl_3_ (d_Cl_), as shown in Figure 6, the measured atomic ratio between Ni and Cl species Ni/Cl allows the molar ratio between Ni_2_O_3_ and FeCl_3_ to be deduced as Ni_2_O_3_/ FeCl_3_=1.5 Ni/Cl. The results are also listed in Table 2.

For sample (5), the results from both the XRD analysis and TEM observations show that the γ-Fe_2_O_3_ grain size is the same and the particles size is less than the samples (1), (2), (3) and (4), and the both sizes are about the same. Since there is much less Ni species than Fe, it is concluded that the spherical particles could consist of a γ-Fe_2_O_3_ core and a Ni_2_O_3_ surface layer. The average particle size depends mainly on the γ-Fe_2_O_3_ phase, and the rod-shaped particles may consist of crystals of 2FeCl_3_·5H_2_O. This is also in agreement with the Fe:Cl ratio for samples (1) to (4) which shows a decrease rather than an increase.

In summary, as the concentration of FeCl_2_ solution used for the chemically induced transition increases, the samples retain a constant γ-Fe_2_O_3_ composition but the proportion of Ni_2_O_3_ is reduced and that of 2FeCl_3_·5H_2_O increases. Clearly, the non-monotonic variation of the specific magnetization of the samples as a function of FeCl_2_ concentration can be attributed to the phase changes. These can be formulated as follows.

The specific magnetization of the samples σ can be described as

(1) $$\sigma =\phi_{m,\gamma }\sigma_{\gamma }+\phi_{m,\mathrm{Ni}}\sigma_{\mathrm{Ni}}+\phi_{m,\mathrm{Cl}}\sigma_{\mathrm{Cl}}$$

where σ_γ_, σ_Ni_, and σ_Cl_ are specific magnetizations, and ϕ_m, γ_, ϕ_m, Νi_ and ϕ_m, Cl_ are the mass fractions of the γ-Fe_2_O_3_, Ni_2_O_3_ and 2FeCl_3_·5H_2_O phases, respectively. According to the definition of the mass fraction, the relationship between ϕ_m, γ_, ϕ_m, Νi_ and ϕ_m, Cl_ is ϕ_m, γ_+ ϕ_m, Νi_ +ϕ_m, Cl_=1. So, formula (1) can be written as

(2) $$\sigma =\sigma_{\gamma }-\left[\phi_{m,\mathrm{Ni}}\left(\sigma_{\gamma }-\sigma_{\mathrm{Ni}}\right)+\phi_{m,\mathrm{Cl}}\left(\sigma_{\gamma }-\sigma_{\mathrm{Cl}}\right)\right]$$

In addition, the ϕ_m, Νi_ and ϕ_m, Cl_ can be described as follows

$$\phi_{m,\mathrm{Ni}}=\frac{\rho_{\mathrm{Ni}}}{\rho_{\mathrm{Ni}}-\rho_{\mathrm{Cl}}+\left[\phi_{v,\gamma }\left(\rho_{\gamma }-\rho_{\mathrm{Cl}}\right)+\rho_{\mathrm{Cl}}\right]/\phi_{v,Ni}}$$

(3) $$\phi_{m,\mathrm{Cl}}=\frac{\rho_{\mathrm{Cl}}}{\rho_{\mathrm{Cl}}-\rho_{\mathrm{Ni}}+\left[\phi_{v,\gamma }\left(\rho_{\gamma }-\rho_{\mathrm{Ni}}\right)+\rho_{\mathrm{Ni}}\right]/\phi_{v,Cl}}$$

where ρ_γ_, ρ_Νi_ and ρ_Cl_ are the densities, and ϕ_v, γ_, ϕ_v, Νi_ and ϕ_v, Cl_ are the volume fractions of γ-Fe_2_O_3_, Ni_2_O_3_ and 2FeCl_3_·5H_2_O, respectively, and *ϕ*_*v*,*γ*_ + *ϕ*_*v*,*Ni*_ + *ϕ*_*v*,*Cl*_ = 1. From the experimental results, it is clear that *ϕ*_*v*,*γ*_ can be regarded as constant for all the samples. Thus, it can be determined from equation (3) that the variations of ϕ_m, Νi_ and ϕ_m, Cl_ depend on ϕ_v, Νi_ and ϕ_v, Cl_, respectively. In addition, the γ-Fe_2_O_3_ is ferrimagnetic, Ni_2_O_3_ is weakly magnetic and 2FeCl_3_·5H_2_O is paramagnetic, so that the magnetization of the samples depends mainly on the γ-Fe_2_O_3_ phase. Therefore, since ϕ_m, Νi_(σ_γ_−σ_Ni_) >>ϕ_m, Cl_(σ_γ_−σ_Cl_), equation (2) can be written as

(4) $$\sigma ≌\sigma_{\gamma }-\left[\phi_{m,\mathrm{Ni}}\left(\sigma_{\gamma }-\sigma_{\mathrm{Ni}}\right)\right]$$

So, for concentrations of FeCl_2_ solution below 0.100 M, as the concentration increases from 0.025 to 0.100 M, the ϕ_m, Νi_ (or ϕ_v, Νi_) decreases gradually, so that σ increases. As long as ϕ_m, Νi_(σ_γ_−σ_Ni_)<<ϕ_m, Cl_(σ_γ_−σ_Cl_), formula (2) can be written as

(5) $$\sigma ≌\sigma_{\gamma }-\left[\phi_{m,\mathrm{Cl}}\left(\sigma_{\gamma }-\sigma_{\mathrm{Cl}}\right)\right]$$

Therefore, for FeCl_2_ solutions above 0.100 M, as the concentration increases from 0.100 to 1.000 M, the values of ϕ_m, Cl_(or ϕ_v, Cl_ ) increase so that σ is reduced. In addition, it can be deduced that when the concentration of FeCl_2_ solution is about 0.100 M, corresponding to sample(3), perhaps ϕ_m, Νi_(σ_γ_−σ_Ni_)≌ϕ_m, Cl_(σ_γ_−σ_Cl_), i.e. ϕ_m, Νi_/ϕ_m, Cl_≌(σ_γ_−σ_Cl_)/(σ_γ_−σ_Ni_), so the specific magnetization σ has its maximum value.

## Conclusion

Using a chemically induced transition, Ni_2_O_3_- γ-Fe_2_O_3_ bioxide composite nanoparticles can be prepared using FeCl_2_ solutions with different concentrations. Using a number of characterization tools, such as VSM, XRD, TEM and XPS, the dependence of the samples on the concentration of the FeCl_2_ solution has been revealed. When the FeCl_2_ concentration was less than 0.500 M, the samples consisted of spherical Ni_2_O_3_- γ-Fe_2_O_3_ particles, about 11 nm diameter, coated with 2FeCl_3_·5H_2_O. When the FeCl_2_ concentration was 0.500 M, the product consisted of both Ni_2_O_3_- γ-Fe_2_O_3_ spherical particles, of about 8 nm size, and 2FeCl_3_·5H_2_O rod-shaped particles. Nevertheless, the size of the γ-Fe_2_O_3_ grains was about the same for all samples. Significantly, the magnetization of the samples exhibited a non-monotonic variation although the ratio between the Ni and Cl species decreased monotonically with the increasing concentration of the FeCl_2_ solution. It was noticed that samples prepared using FeCl_2_ solutions with concentrations 0.025 M 0.075 M, 0.100 M and 0.125 M, have the same size particles, about 11 nm, and same size of γ-Fe_2_O_3_ grains, about 8 nm. Therefore, it is deduced that the variation of the apparent magnetization has resulted from the competition between the reduced Ni_2_O_3_ phase and increasing 2FeCl_3_·5H_2_O. When the concentration of FeCl_2_ solution does not exceed 0.100 M, the magnetization of the samples increases with increasing concentration since the rate of reduction of Ni_2_O_3_ is larger than the increase of 2FeCl_3_·5H_2_O. When the FeCl2 concentration exceeds 0.100 M, the magnetization of the samples weakens since the increase of 2FeCl_3_·5H_2_O is now larger than the decrease of Ni_2_O_3_. Therefore, it can be concluded that using the chemically induced transition method to prepare Ni-Fe bioxide composite nanoparticles, as long as the concentration of the FeCl_2_ solution does not exceed 0.100 M, the thickness of both Ni_2_O_3_ and 2FeCl_3_·5H_2_O layers can be controlled and the γ-Fe_2_O_3_ core size remains constant. As a result, magnetic nanoparticles with a fixed size of about 11nm but different magnetization can be obtained.

## Competing interests

Non-financial competing interests.

## Authors’ contributions

All authors contributed equally to this work. All authors read and approved the final manuscript.

## References

[B1] Pankhrust QA Connolly J Jones SK Dobson J Application of magnetic nanoparticles in biomedicine J. Phys. D 2003 36 R167 R181 10.1088/0022-3727/36/13/201

[B2] Willard MA Kurihara LK Carpenter EE Calvin S Harris VG Chemically prepared magnetic nanoparticles Inter 2004 49 3/4 125 170

[B3] Sun S Recent advance in chemical synthesis, self-assembly, and applications of FePt nanoparticles Adv Mater 2006 18 393 403 10.1002/adma.200501464

[B4] Lin CR Wang CC Chen IH Magnetic behavior of core-shell particles J 2006 304 e34 e36 10.1016/j.jmmm.2006.02.035

[B5] Jiang J Yang YM Facile synthesis of nanocrystalline spinel NiFe_2_O_4_ via a novel soft chenistry route Mater Lett 2007 61 4276 4279 10.1016/j.matlet.2007.01.111

[B6] Szabo DV Vollath D Nanocomposites from coated nanoparticles Adv Mater 1999 11 1313 1316 10.1002/(SICI)1521-4095(199910)11:15<1313::AID-ADMA1313>3.0.CO;2-2

[B7] Liu J Qiao SZ Hu QH Lu GQ Magnetic nanocomposites with mesoporous structures: synthesis and application Small 2011 7 425 443 10.1002/smll.201001402 21246712

[B8] Lu Y Yin Y Mayers BT Xia Y Modifying the surface properties of superparamagnetic iron oxide nanoparticles through a sol–gel approach NanoLett 2002 2 183 186 10.1021/nl015681q

[B9] Donselaar LN Philips AP Suurmoned J Concentration-dependent sedimentation of dilute magnetic fluids and magnetic silica dispersions Langmuir 1997 13 6018 6025 10.1021/la970359+

[B10] Duarte MA Giersig M Kotov NA Marzan LML Control of packing order of self-assembed monolayers of magnetic nanoparticles with and without SiO2 coating by microwave irradiation Langmuir 1998 14 6430 6435 10.1021/la9805342

[B11] Butterworln MD Illum L Davis SS Preparation of ultrafine silica-and-PEG-coated magnetic particles Colloid Sur. A 2001 179 93 102 10.1016/S0927-7757(00)00633-6

[B12] Kawaguchi K Jaworski J Ishikawa Y Sasaki T Koshizaki N Preparation of gold/iron-oxide composite nanoparticles by a unique laser process in water J 2007 310 2369 10.1016/j.jmmm.2006.11.109

[B13] Yuvaraj H Woo MH Park EJ Jeong YT Lim KT Polypyrrole/γ-Fe_2_O_3_ magnetic nanocomposites synthesized in supercitical fluid Eur Polym J 2008 44 637 644 10.1016/j.eurpolymj.2008.01.007

[B14] Xu XJ Wang J Yang CQ Wu HY Yang FF Sol–gel formation of γ-Fe_2_O_3_/SiO_2_ nanocomposites: Effects of different iron raw material J Alloys Compd 2009 468 414 420 10.1016/j.jallcom.2008.01.013

[B15] Zhang QM Li J Lin YQ Liu YD Miao H The spreparation and characterization of NI-Fe bioxide composite nanoparticles J 2010 508 396 399 10.1016/j.jallcom.2010.08.065

[B16] Zhang QM Li J Miao H Fu J Preparation of γ-Fe_2_O_3_/Ni_2_O_3_/FeCl_3_(FeCl_2_) composite nanoparticles by hydrothermal process useful for ferrofluids Smart Mater Res 2011 (page number not for citation purposes) 10.1155/2011/35/072

[B17] Liu Q Xu Z Finch JA Egerton R A novel two-step silica-coating process for engineering magnetic nanocomposites Chem Mater 1998 10 3936 3940 10.1021/cm980370a

[B18] Wen BC Li J Lin YQ Liu XD Fu J Miao H Zhang QM A novel preparation method for γ-Fe_2_O_3_ nanoparticles and their characterization Mater 2011 128 35 38 10.1016/j.matchemphys.2011.01.012

[B19] Miao H Li J Lin YQ Liu XD Zhang QM Fu J Characterization of γ-Fe_2_O_3_ nanoparticles prepared by transformation of α-FeOOH Chinese Sci 2011 56 2383 2388

[B20] Lin LH Li J Fu J Lin YQ Liu XD Preparation, magnetization, and microstructure of ionic ferrofluids based on γ-Fe_2_O_3_/Ni_2_O_3_ composite nanoparticles Mater 2012 134 407 411 10.1016/j.matchemphys.2012.03.009

[B21] Li J Lin YQ Liu XD Zhang QM Miao H Zhang TZ Wen BC The study of transition on NiFe_2_O_4_ nanoparticles prepared by co-precipitation/calcinations Phase Trans 2011 84 49 57 10.1080/01411594.2010.521432

[B22] Iwacaki T Kosaka K Watano S Yanagida T Kawai T Novel environmentally friendly synthesis of superparamagnetic magnetite nanoparticles using mechanochemical effect Mater Res Bull 2010 45 481 485 10.1016/j.materresbull.2009.11.006

[B23] Srnová-Šloufová I Vlcková B Bastl Z Hasslet TL Bimetallic (Ag) Au nanoparticles prepared by the seed growh: Two-dimensional assembling, characterization by energy disperse X-ray analysis X-ray photoelectron spectroscopy, and surface enhanced Raman Spectroscopy, and proposed mechanism of growth, Langmuir 2004 20 3407 3415 10.1021/la0302605 15875875

[B24] Tanuma S Powell CJ Penn DR Calculations of electron inelastic mean free paths Surf Interface Anal 1991 17 911 926 10.1002/sia.740171304 PMC5524379 28751796

